# BRAF Mutations in Canine Cancers

**DOI:** 10.1371/journal.pone.0129534

**Published:** 2015-06-08

**Authors:** Hiroyuki Mochizuki, Katherine Kennedy, Susan G. Shapiro, Matthew Breen

**Affiliations:** 1 Department of Molecular Biomedical Sciences, College of Veterinary Medicine, North Carolina State University, Raleigh, North Carolina, United States of America; 2 Center for Comparative Medicine and Translational Research, North Carolina State University, Raleigh, North Carolina, United States of America; 3 Center for Human Health and the Environment, North Carolina State University, Raleigh, North Carolina, United States of America; 4 Lineberger Comprehensive Cancer Center, University of North Carolina, Chapel Hill, North Carolina, United States of America; Ohio State University Medical Center, UNITED STATES

## Abstract

Activating mutations of the *BRAF* gene lead to constitutive activation of the MAPK pathway. Although many human cancers carry the mutated *BRAF* gene, this mutation has not yet been characterized in canine cancers. As human and canine cancers share molecular abnormalities, we hypothesized that *BRAF* gene mutations also exist in canine cancers. To test this hypothesis, we sequenced the exon 15 of *BRAF*, mutation hot spot of the gene, in 667 canine primary tumors and 38 control tissues. Sequencing analysis revealed that a single nucleotide T to A transversion at nucleotide 1349 occurred in 64 primary tumors (9.6%), with particularly high frequency in prostatic carcinoma (20/25, 80%) and urothelial carcinoma (30/45, 67%). This mutation results in the amino acid substitution of glutamic acid for valine at codon 450 (V450E) of canine *BRAF*, corresponding to the most common *BRAF* mutation in human cancer, V600E. The evolutional conservation of the *BRAF* V600E mutation highlights the importance of MAPK pathway activation in neoplasia and may offer opportunity for molecular diagnostics and targeted therapeutics for dogs bearing *BRAF*-mutated cancers.

## Introduction

The RAF proteins are evolutionary conserved serine/threonine kinases that regulate fundamental cellular processes, including growth, differentiation and survival. The RAF family consists of three members: ARAF, BRAF and CRAF. All RAF proteins are activated by RAS and subsequently activate MEK, initiating the signal transduction cascade of the MAPK pathway. Constitutive activation of the MAPK pathway caused by oncogenic mutations of *RAF* genes results in abnormal proliferation and differentiation. Among the three forms of *RAF* genes, *BRAF* gene is most frequently mutated in human cancer [1–3].

The most common (>90%) somatic mutation of the human *BRAF* gene is a T-to-A transversion in exon 15 at nucleotide 1799 (c.1799T>A), resulting in the amino acid substitution from valine to glutamic acid at codon 600 (V600E) [2]. The V600E mutation occurs within the activation segment of the gene and mimics phosphorylation, drastically elevating kinase activity and activation of the downstream signal [3,4]. This activating mutation has been reported in melanoma (~60%) [4,5], thyroid cancer (20–40%)[6–9], hairy-cell leukemia (~100%)[10] and many other cancers with variable frequency. Coupled with frequent mutations of *RAS* genes, the presence of *BRAF* mutations in a wide variety of human cancers underscores the importance of MAPK pathway activation as a common oncogenic molecular pathway.

Dogs develop spontaneous cancers with many similarities to human cancers, including anatomical location, histological appearance and therapeutic response. Cancer in dogs shares not only biological behaviors with humans, but also molecular abnormalities [11,12]. Since activating *BRAF* mutations are present in a wide variety of human cancer, we hypothesized that *BRAF* gene mutations are similarly involved in canine cancers, leading to hyperactivation of the MAPK pathway and cell transformation. To test this hypothesis, we screened for the presence of *BRAF* exon 15 mutations in a cohort of 667 pathologically confirmed canine tumor specimens, comprising a series of hematopoetic tumors (n = 245), sarcomas (n = 160), carcinomas (n = 115), melanocytic tumor (n = 72) as well as other, less common cancer (n = 75).

## Materials and Methods

Fresh and formalin fixed tumor specimens of various canine solid tumors, and EDTA blood samples from canine leukemia cases, were submitted from client-owned pet dogs (with informed owner consent) by private veterinary practices across the United States as a part of routine diagnostic procedures (no IACUC required). Additional tumor specimens were recruited via the North Carolina State University (NCSU) Clinical Studies Core, each with informed owner consent and following an NCSU IACUC approved protocol (approval number 13-022-O), which covered the procedure used to obtain the samples and their subsequent use for research application. Hematoxylin and eosin-stained slides of formalin-fixed paraffin-embedded (FFPE) specimens were reviewed by a board-certified veterinary pathologist and confirmed neoplastic in all but leukemia specimens. Leukemia diagnoses were based on the evaluation of cytological and immunophenotypical examination of leukemic cells by a board-certified clinical pathologist.

Genomic DNA was isolated from fresh tissue/blood samples or FFPE tissues. A total of 667 tumor specimens were included in this study. Details of the sample population are shown in Tables 1 and 2. DNA was isolated using a QIAamp FFPE DNA extraction kit (Qiagen, Valencia, CA, USA) or a DNeasy Blood and Tissue Kit (Qiagen). Spectrophotometry (NanoDrop, Thermo Scientific, Wilmington, DE) and agarose gel electrophoresis were used to determine DNA quantity and integrity. For non-neoplastic controls (n = 38), DNA was isolated from canine bladder epithelium of 30 dogs and prostate glands of 8 dogs, obtained by necropsy with no evidence of neoplastic changes upon histopathologic evaluation.

**Table 1:** Primary cancer samples used in this study.

Cancer type	N	Pathological classification
Hematopoietic	245	Lymphoma (50), mast cell tumor (50), chronic lymphocytic leukemia (43; 20 B-cell and 23 T-cell origin), histiocytoma (27), plasmacytoma (21), histiocytic sarcoma (20), acute myelgenous leukemia (18), acute lymphoblastic leukemia (16)
Sarcoma	160	Soft tissue sarcoma (60), hemangiosarcoma (50), osteosarcoma (50)
Carcinoma	115	Urothelial carcinoma (45), prostatic carcinoma (25), pulmonary carcinoma (18), oral squamous cell carcinoma (18), mammary gland carcinoma (7), anal sac carcinoma (1), renal cell carcinoma (1)
Melanocytic	72	Melanoma (54; 47 oral, 6 cutaneous and 1 ocular origin), melanocytoma (18)
Miscelleneous	75	Meningioma (20), ameloblastoma (16), transmissible venereal tumor (14), glioma (13), peripheral nerve sheath tumor (9), nephroblastoma (3)

**Table 2:** Signalments of dogs diagnosed with primary cancers.

Breed	Mixed (83), Labrador Retriever (82), Golden Retriever (72), Boxer (38), German Shepherd Dog (28), Beagle (18), Flat-Coated Retriever (17), Greyhound (17), Australian Shepherd (16), Pug (12), Bernese Mountain Dog (10), Miniature Shnauzer (10), other breeds (< 10 each, 223)
Gender	Male (347), female (311), unknown (9)
Neutering	Castrated (238), spayed (230), intact (8), unknown (221)
Age	< 3 years old (15), 3–7 years old (143), 8–11 years old (248), 11 < years old (124), unknown (137)
DNA source	FFPE tissue (458), fresh frozen tissue (132), blood (77)

**Fig 1:**
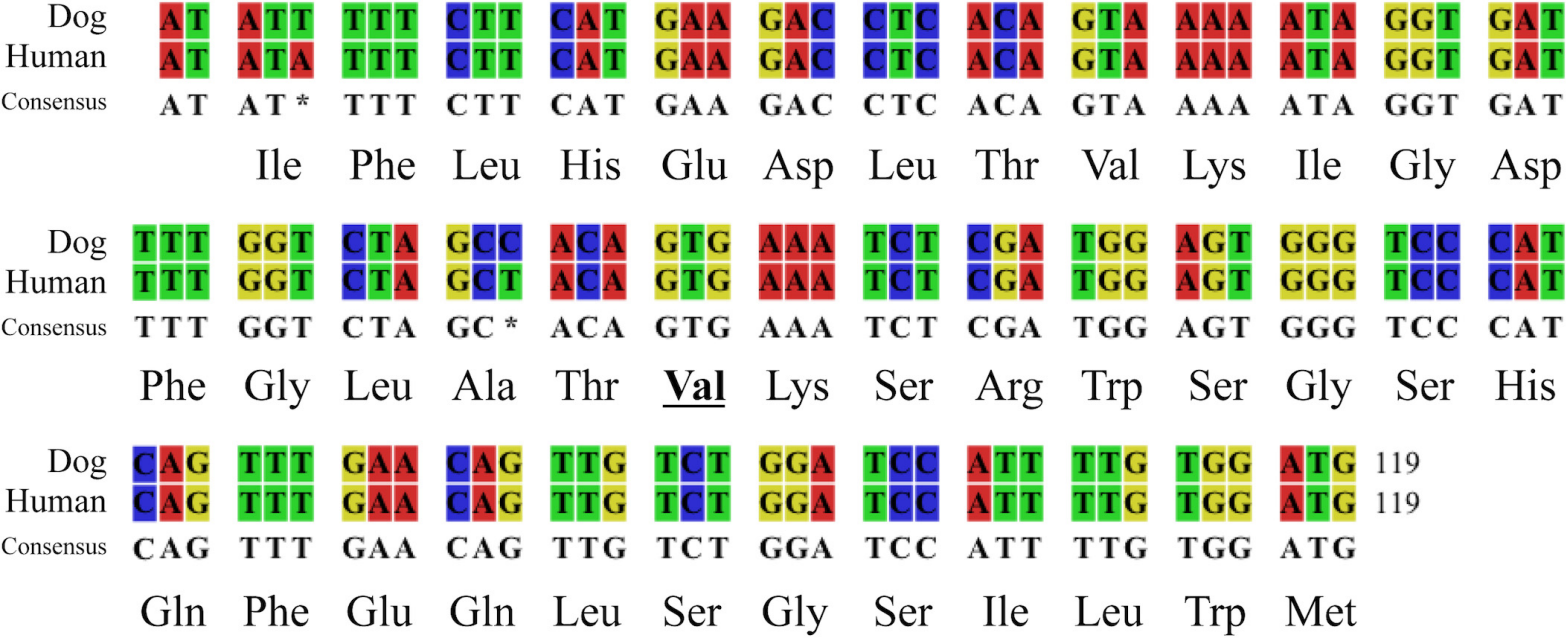
DNA and amino acid sequences of human (NM_004333) *BRAF* exon 15 and dog *BRAF* gene (XM_005629550.1). The sequences are highly conserved between human and dog, including valine at codon 600 in human BRAF (underlined) and at codon 450 in canine BRAF.

Exon 15 of the human *BRAF* gene is evolutionally conserved between dogs and humans (Fig 1). Thus, PCR amplification was performed to amplify a 391-bp DNA fragment spanning the genomic canine *BRAF* sequence corresponding to human *BRAF* gene exon 15 (CanFam3.1, canine chromosome (CFA) 16: 8,296,227–8,296,345). The following primer pair was designed using Primer-BLAST software (http://www.ncbi.nlm.nih.gov/tools/primer-blast/): forward, AAGCAGGTCACATATGCCAAA (CFA 16: 8,296,007–8,296,027); reverse, ATTTTTGGACCCTGAGGTGC (CFA 16: 8,296,378–8,296,397). Each PCR reaction contained 10–20 ng of genomic DNA, 250 nM of the forward and reverse primer and 1× Taq RED Master Mix Kit (Genesee Scientific, San Diego, CA, USA). PCR cycles consist of initial denaturation of 95°C for 2 min, followed by 40 cycles of 95°C for 30 s, 60°C for 30 s and 72°C for 30 s with a final elongation step at 95°C for 5 min. PCR products were visualized using agarose gel electrophoresis and subjected to targeted Sanger sequencing analysis with the forward and/or reverse primers. Sequence analysis was performed at the North Carolina State University Genome Research Laboratory (http://research.ncsu.edu/gsl/). The sequencing data were analyzed using 4peaks software (http://nucleobytes.com/index.php/4peaks) and compared with the reference sequence (XM_005629550.1) using CLC Sequence Viewer version 7 (CLC bio, Aarthus, Denmark).

The Fisher’s exact test was performed to examine difference of *BRAF* mutation frequencies between groups stratified by gender, neutering status, age or breeds. All statistical analyses were performed with JMP Pro software version 11 (SAS Institute, Cary, NC). Values of P < 0.05 were considered significant.

## Results and Discussion

**Fig 2:**
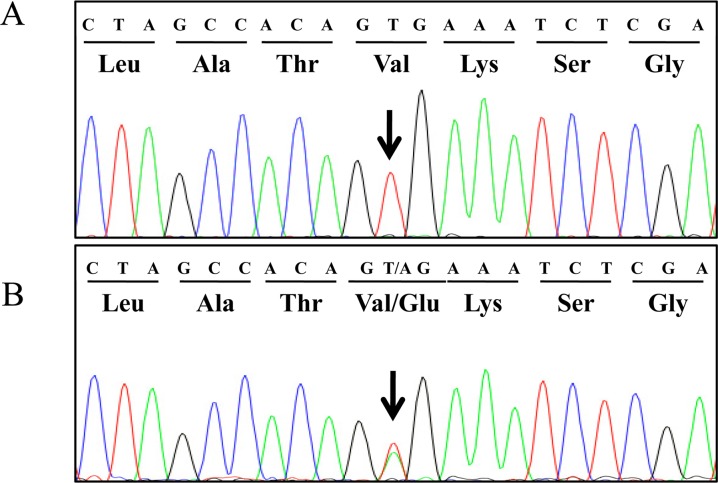
Sequence analysis of the canine BRAF gene. (A) Wild-type sequence obtained from a control prostate gland DNA. (B) Mutated sequence mixed with wild-type sequence obtained from a prostatic carcinoma. Arrow indicates the T-to-A nucleotide substitution resulting in the change of valine at codon 450 to glutamic acid.

To investigate the presence of *BRAF* mutations, we sequenced *BRAF* gene exon 15 in 667 primary tumor samples and 38 control tissue samples. Sequencing analysis revealed a T to A transversion at nucleotide 1349 (c.1349T>A, reference: XM_005629550.1) which occurred in 64 primary tumors, resulting in the amino acid substitution from valine to glutamic acid at codon 450 (V450E) (Fig 2). This amino acid change corresponds to the human V600E mutation (Fig 1). Significant variation exists in the frequency of the V450E mutation across canine cancers: 0% in hematopoietic tumors and sarcomas to 67% and 80% of urothelial carcinoma (UC) and prostatic carcinoma (PC), respectively (Table 3). In all V600E mutants, electropherograms indicated the presence of both mutated and wild-type sequences, suggesting mutation heterozygosity. There was no statistically significant difference in the mutation frequency between different groups of neutering status, age or breeds in UC and PC samples and between the mutational status and gender in UC samples. Details of signalments of dogs with UC and PC are shown in S1 Table.

**Table 3:** Prevalence of V450E mutation in canine primary cancers.

Cancer type		N	V450E (frequency)
Hematopoietic	Lymphoma	50	0
	Mast cell tumor	50	0
	Chronic lymphocytic leukemia	43	0
	Histiocytoma	27	0
	Plasmacytoma	21	0
	Histiocytic sarcoma	20	0
	Acute myelogenous leukemia	18	0
	Acute lymphoblastic leukemia	16	0
Sarcoma	Soft tissue sarcoma	60	0
	Hemangiosarcoma	50	0
	Osteosarcoma	50	0
Carcinoma	Urothelial carcinoma	45	30 (67%)
	Prostatic carcinoma	25	20 (80%)
	Pulmonary carcinoma	18	1 (6%)
	Oral squamous cell carcinoma	18	2 (11%)
	Other carcinoma	9	0
Melanocytic	Melanoma	54	3 (6%)
	Melanocytoma	18	3 (17%)
Miscellaneous	Meningioma	20	0
	Ameloblastoma	16	0
	Transmissible venereal tumor	14	0
	Glioma	13	2 (15%)
	Peripheral nerve sheath tumor	9	2 (22%)
	Nephroblastoma	3	0

In addition to the V450E mutation, a T-to-C transition at nucleotide 1305 (c.1305T>C, silent mutation) was observed in an oral squamous cell carcinoma sample. An intronic deletion of T (c.1292-189delT) was observed in one each of soft tissue sarcoma and melanoma samples. All other tumor and control samples maintained the wild type genomic sequence for *BRAF* exon 15.

Constitutive activation of MAPK signaling by activating mutations of *BRAF* (~60%) or *NRAS* (~15%) genes plays an important role in the pathogenesis of human melanoma [4,5,11,12]. Similarly, constitutive activation of the MAPK pathway is implicated in canine melanoma [13,14], although *RAS* genes were infrequently mutated [14–16]. In this study, however, only 6% of melanomas (two mucosal and one cutaneous melanoma) and 17% of melanocytomas harbored the *BRAF* V450E mutation. This mutation was not identified in previous studies of canine melanoma [13,17], likely due the low frequency of the *BRAF* mutation in canine melanoma. In human melanoma, the presence of *BRAF* mutation is associated with skin exposure to UV light, and melanomas on mucosal sites or non-UV-exposed skin rarely possess the mutation [18,19]. As canine melanoma occurs mainly on oral mucosa and infrequently on nail beds and non-UV-exposed furred skin, the fact that *BRAF* is mutated infrequently in canine melanoma is consistent with findings in human counterparts.

Interestingly, canine UC showed much higher frequency of the *BRAF* mutation than is reported to in human UC tumors [20]. Mutations in genes upstream of the MAPK pathway, including *HRAS*, *KRAS* and *FGFR3* genes (all of which are upstream molecules of BRAF in MAPK pathway), were found in >82% of human papillary UC, suggesting that activation of the pathway is a main driving factor for the subclass of human UC [21,22]. Although the mutated molecules in the pathway may be different between human and canine UC, the high frequency of *BRAF* mutation in canine UC suggests that dysregulation of MAPK pathway may play an important role in the pathogenesis of the disease.

Canine PC is characterized by high metastatic potential and local invasiveness, but the factors contributing to aggressive biological behavior are still largely unknown [23]. Although *BRAF* V600E mutations are infrequent in humans [24–26], accumulating evidence suggests that MAPK pathway plays an important role in the development and progression of human PC, especially in metastatic tumors [27]. Somatic mutations of the *RAS* genes and copy number gains of *BRAF* and *CRAF* genes are observed in human PC at frequencies of ~10, 30% and 15%, respectively [24–27]. These genomic alterations lead to the activation and/or increased expressions of RAF proteins, resulting in the activation of downstream signaling and increasing metastatic properties [26–28]. Additionally, recurrent chromosomal translocations involving *RAS* and *RAF* genes, which result in oncogenic fusion genes, were recently discovered in a subset (~5%) of human PC cases [29,30].

A unique feature of canine PC is that the majority of tumors arise in androgen-independent manner, with increased risk in castrated dogs [23]. On the other hand, hormone-deprivation therapy is a mainstay for the treatment of human PC, as androgen plays a critical role in the pathogenesis. Most of human PC, however, progress to a more aggressive, hormone-refractory (castration-resistant) cancer during the clinical course. Activation of BRAF/MAPK signaling makes human PC tumor cells less dependent on androgen for proliferation *in vivo* and *in vitro*, contributing to hormone-refractory phenotype [31]. The high incidence of the *BRAF* mutation and aggressive nature of canine PC may reflect the fact that most canine PC develop independently of androgen stimulation. These clinical and molecular similarities may make canine PC serve as a spontaneously-occurring animal cancer model relevant to hormone-refractory human PC.

Recent advancement in molecular technology enabled us to detect circulating tumor cells in liquid samples such as peripheral blood. Detection of *BRAF* mutations can be used as a means to diagnose and monitor tumor burden in liquid samples, such as blood or urine, without necessitating biopsy of tumors (called as liquid biopsy, reviewed in [32,33]). Although histopathological examination of a tumor biopsy is the gold standard for the diagnosis of canine UC and PC, the anatomical locations of these tumors often make it difficult to obtain sufficient amount of tissues to diagnose. Additionally, clinicians and owners may be discouraged from choosing this diagnostic workup due to cost and the invasive procedures associated with biopsy. Therefore, access to a non-invasive means of diagnosing these cancers is an unmet need. The high *BRAF* mutation rate in these tumors makes the BRAF V450E mutation a potential diagnostic marker for affected cancers.

The identification of *BRAF* mutation in canine cancers raises the possibilities that therapy targeting constitutively-activated MAPK pathway can provide a clinical benefit for those carrying the *BRAF* V450E mutation, especially UC and PC patients. Recently, vemurafenib and dabrafenib, selective BRAF inhibitors, improved clinical outcomes in patients with melanoma compared to conventional chemotherapy [34,35]. These BRAF inhibitors have also shown therapeutic potentials in other neoplasms harboring *BRAF* mutations [36–38]. Currently, treatment options for dogs with UC and PC are of limited efficacy. Given the effectiveness of BRAF/MAPK-targeted therapy in human cancers, the BRAF and MAPK pathways may be promising therapeutic targets for these canine cancers. Evaluations of *in vitro* and *in vivo* effects of BRAF inhibitors in dogs are warranted for the clinical application of the BRAF inhibitor for dogs bearing cancer with mutated *BRAF*.

In conclusion, we identified the *BRAF* V450E mutation in canine cancers with various frequencies. Frequent *BRAF* mutation in canine UC and PC underscores a potential role of the MAPK signaling pathway in the pathogenesis of these tumors and may offer diagnostic and therapeutic applications for dogs bearing *BRAF* mutations.

## Supporting Information

S1 Table Signalments of dogs diagnosed with urothelial and prostatic carcinoma in the present study. (XLSX)
